# Vitamin D knowledge and sun exposure practices among Sri Lankan healthcare undergraduates

**DOI:** 10.1371/journal.pone.0279480

**Published:** 2022-12-27

**Authors:** Guwani Liyanage, Sanjana Jayathunga, Thamara Amarasekara

**Affiliations:** 1 Department of Paediatrics, Faculty of Medical Sciences, University of Sri Jayewardenepura, Nugegoda, Sri Lanka; 2 Department of Nursing, Faculty of Allied Health Sciences, University of Sri Jayewardenepura, Nugegoda, Sri Lanka; Endocrinology and Metabolism Population Sciences Institute, Tehran University of Medical Sciences, ISLAMIC REPUBLIC OF IRAN

## Abstract

**Introduction:**

Although overexposure to ultraviolet radiation may lead to skin cancer, inadequate exposure results in vitamin D deficiency (VDD). We explored vitamin D-related knowledge and sun exposure practices among Sri Lankan healthcare undergraduates.

**Methods:**

The sampling frame consisted of medical and allied health undergraduates in a single centre. A newly developed, pre-piloted, self-administered questionnaire collected data on vitamin D knowledge, sun avoidance behaviour and outdoor time. Univariate and multivariate logistic regression analysis examined the factors related to outdoor time.

**Results:**

A total of 482 were included in the analysis. The mean (SD) vitamin D knowledge score (0–100% scale) was 31.3% (18%). Only 17.8% scored ≥50% for knowledge. At least one sun avoidance measure was used by 59.3% of the undergraduates. A lower knowledge score was observed with a higher number of sun-avoidance behaviour (mean difference 0.84, p = 0.03). The majority (66%) spent outdoors <30 minutes per day between 9 am-3 pm. The odds of having low outdoor time were 1.6 higher for the female sex (OR:1.61, 95%CI:1.039, 2.492, p<0.001) and studying in the final year (OR:1.63, 95%CI:1.020, 2.602, p = 0.04). Medical students had a higher likelihood of low outdoor time (OR:0.55, 95%CI: 0.361, 0.835, p = 0.005).

**Conclusions:**

The healthcare undergraduates had low vitamin D knowledge and outdoor time while having increased sun avoidance. Gender, course of study, and academic year appeared to affect outdoor time. Support and guidance should improve knowledge and sun exposure habits that suit academic work and lifestyle in this population. Also, universities can actively promote positive sun exposure by organizing outdoor events.

## Introduction

Vitamin D plays a significant role in skeletal health. Also, there is a plethora of evidence for many non-skeletal functions [1]. Especially in the era of the overwhelming COVID-19 disease burden, the benefits of vitamin D have been promising [2].

Countries with abundant sunshine have reported a high prevalence of vitamin D deficiency [3]. Therefore, the notion that living in tropical countries would ensure adequate vitamin D can no longer be accepted. Sri Lanka is located at a latitude of 6° 55’ and has a tropical climate (average temperature 27.5°C) with plentiful sunshine throughout the year [4]. Yet, several cross-sectional studies have reported vitamin D deficiency among children and adults in Sri Lanka [5, 6]. Subasinghe et al. reported a cumulative prevalence of vitamin D deficiency and insufficiency of 90.2% in an urban community sample of 18 years and above. The highest prevalence was observed among younger than older people [6].

Reasons for vitamin D deficiency (VDD) in people living in tropical countries may be manifold: busy lifestyles, low outdoor activities, and sedentary behaviour patterns [7]. According to Webb et al., Asians with dark skin require approximately 30 minutes of sun exposure for adequate vitamin D [8]. Sun exposure was exceptionally low during the COVID-19 pandemic because people spent more time indoors with frequent lockdowns [9]. Further, food fortification and vitamin D supplementation programs are almost non-existent in most countries [10].

There are mixed messages and recommendations regarding the benefits and harms of sun exposure. Therefore, it is not surprising that most are confused about the safe level of sun exposure [11]. Also, there are contrasting reports on vitamin D knowledge of health care workers and undergraduates in different populations [12, 13]. In the Sri Lankan context, the evidence of sun exposure practices and vitamin D knowledge among healthcare undergraduates is currently unknown. Therefore, understanding undergraduates’ vitamin D knowledge, sun avoidance practices, and outdoor time could help to guide interventions aimed at vitamin D-related health.

## Methods

### Study design and procedure

The sampling frame comprised the entire undergraduate cohort (nursing, pharmacy, and medical laboratory science) of the last three academic years (n = 598) at a university for medical sciences in Sri Lanka. They all have had nutrition lectures during the foundation year. The sample size was calculated with an expected frequency of knowledge score >50% as 58% [14], with a 95% confidence interval and a 5% margin of error. After correction for dropouts (20%), the minimum calculated sample size was 448. The students with vitamin D-related illnesses were excluded to select a homogenous sample. The questionnaire was disseminated through the university e-mail list from January to March 2020, inviting to participate in the anonymous survey. All respondents who gave written informed consent answered the questionnaire on a Google platform. Ethics clearance was obtained from the Ethics Review Committee of the Faculty of Medical Sciences, University of Sri Jayewardenepura (ERC-NUR19/20).

### Study instrument

Based on previous research, the self-administered questionnaire was designed specifically for this study [14]. Two physicians who are experts in the field examined the content and item construction of the questionnaire. Also, questionnaires were pre-tested among 12 students to ensure that questions were unambiguous, clear, and easy to understand.

The first section collected data on demography and height and weight reported by the respondent. They were asked to indicate any physician-diagnosed vitamin D-related illness if any. Eight multiple-choice questions assessed vitamin D knowledge. Two questions (knowledge of risk factors for vitamin D deficiency and best natural food sources of vitamin D) expected multiple answers, and six remaining questions expected one answer. Three questions (using sunscreen, wearing protective clothing/umbrella, or seeking shade when going outside) assessed sun-avoidance behaviour, answered on a 5-point Likert scale (always = 4, often = 3, sometimes = 2, rarely = 1, and never = 0). The percentages of undergraduates who reported that they always/often practice protective measures to avoid the sun (using sunscreen, wearing protective clothing/umbrella, or seeking shade when going outside) were computed. Reasons for sun avoidance (skin darkening and skin cancer risk) were explored on the same 5-point Likert scale above. The reported outdoor time between 9 am to 3 pm was documented. Outdoor time was dichotomized (≥30 minutes vs <30 minutes) based on previous reports on adequate sun exposure for sufficient vitamin D [8].

### Data analysis

The data were analyzed using IBM® SPSS® Statistics Version 22.0. Quantitative variables are expressed as mean with standard deviation or median with interquartile range (IQR). Each vitamin D knowledge question was given equal weight (three points). The questions with one correct answer received three or else zero. For the questions with more than one correct answer, each correct answer was awarded equally weighted points within that question. Equally weighted points were subtracted if the respondent had marked incorrect responses. Also, the response "I do not know’’ was awarded zero for one or multiple answer questions. A Vitamin D knowledge score of ≥50% was considered satisfactory.

Body mass index (BMI) was calculated with the reported weight and height. BMI was categorized as underweight (<18kg/m2), normal (18–24.9 kg/m2) and overweight/obese (≥25kg/m2) [15]. The independent sample t-test was used for bivariate comparisons. Sun-avoidance behaviour was assessed as a percentage of undergraduates who reported that they often or always practised each kind of sun protection behaviour. Reasons for sun avoidance reported by the respondents were dichotomized (always/often vs sometimes/rarely/never) and were expressed as percentages. Simple and multivariate logistic regression analysis evaluated factors related to outdoor time (dependent variable). With prior knowledge [16, 17], the following independent variables were considered for simple linear regression: course of study (medical vs allied health), final year/not, gender, age, BMI, vitamin D knowledge, and sun avoidance measures (using at least one sun avoidance measure vs no measure). After checking for assumptions, all variables with p<0.1 were included in the multivariate logistic regression analysis.

## Results

A total of 593 students were invited. Four hundred and eighty-five responded. Two medical undergraduates (MU) and one nursing undergraduate (NU) were excluded as they had vitamin D-related illnesses. The final sample consisted of 482 (medical = 339, nursing = 48, pharmacy = 45, and medical laboratory science = 50), and the response rate was 82%. The median age (IQR) was 25 years (24, 26). Female respondents were predominant (n = 356, 73.9%). The majority were final-year students (52%). BMI’s median (IQR) was 21.9 kg/m^2^ (19.7, 24.2) and 16.6% (n = 80) were overweight/obese, and 8.5% were underweight. Almost half (44%) were concerned about their vitamin D level. Only 5% reported using vitamin D supplements.

### Vitamin D knowledge

The mean (SD) knowledge score (scale of 0–100%) was 31.3% (18). Only 17.8% (n = 86) scored ≥50%. There was no statistically significant difference in knowledge in terms of gender [*F* (1,481) = 3.78, *p* = 0.05], academic year [F (1,481) = 1.01, p = 0.31] and study course (medical/allied health) [F (1,481) = 0.36, p = 0.55]. Table 1 shows the participants’ vitamin D knowledge based on eight questions.

**Table 1:** Participants’ vitamin D knowledge based on eight questions.

	Correctly answered (n)	%
The best source of vitamin D (sunlight)	293	60.8
Type of UVR required for vitamin D production (UVB)	106	22.0
Best time for sun exposure (9 am-3 pm)	132	27.4
Sitting indoors by a plain glass window is adequate (no)	167	34.6
Risk factors for vitamin D deficiency		
Not going outdoors (√)	369	76.6
Covered clothing (√)	276	57.3
Dark skin (√)	130	27.0
Not eating fish (×)	247	51.2
Dairy allergy (×)	337	69.9
None of the above (×)	473	98.1
Best natural food sources of vitamin D		
Fish (√)	325	67.4
Fresh milk (×)	259	53.7
Pulses (×)	375	77.8
Vegetables (×)	329	68.3
Fruits (×)	368	76.3
Eggs (√)	274	56.8
Vitamin D plays a role in non-skeletal health (yes)	149	30.9
Vitamin D prevents rickets (yes)	432	89.6

Correct answers are given within brackets.

Abbreviations: UVR-Ultraviolet rays, UVB-Ultraviolet B

Less than 50% of the participants correctly answered five knowledge questions: type of ultraviolet rays (UVR) required for vitamin D synthesis, the best time for sun exposure, ultraviolet B (UVB) ray penetration through plain glass, risk factors for vitamin D deficiency (i.e., dark skin), and the role of vitamin D in non-skeletal health (Table 1).

### Sun avoidance

The sun avoidance practices while being outdoors were explored (Table 2). At least one sun avoidance measure was used by 59.3% of the undergraduates. The most frequent sun avoidance measure was seeking shade (40.5%, 195/482). The use of these measures (at least one measure vs no measure) was not significantly different by course of study (p = 0.7) or gender (p = 0.32). The undergraduates who did not practice any sun avoidance measure had higher knowledge than those who practised at least one measure (mean difference = 0.84, p = 0.03). Reasons for practising sun avoidance were explored; 53.1% did so due to skin darkening, while 17.6% were due to fear of skin cancer.

**Table 2:** Sun avoidance behaviour of the respondents.

	Response	n	%
Seek shade	Always	29	6.0
	Often	166	34.4
	Sometimes	244	50.6
	Rarely	27	5.6
	Never	16	3.3
Use sunscreen	Always	03	0.6
	Often	61	12.7
	Sometimes	250	51.9
	Rarely	160	33.2
	never	08	1.6
Use protective clothing/umbrella	Always	32	6.6
	Often	120	24.9
	Sometimes	150	31.1
	Rarely	108	22.4
	Never	72	14.9

### Outdoor time

Most (66%) spent outdoors <30 minutes/day between 9 am-3 pm. The factors related to outdoor time (<30 minutes vs ≥30 minutes) were explored using logistic regression. Vitamin D knowledge score, age, BMI, academic year, sun-avoidance measures (at least one measure vs no measure), and supplement use were not significant variables in univariate analysis (Table 3).

**Table 3:** Factors associated with outdoor time using univariate logistic regression.

	Exp (B)	95% C. I for B		P-value
		Lower Bound	Upper Bound	
Age	1.08	0.968	1.207	0.17
Sex				
Male	Ref			
Female	1.46	0.961	2.25	0.08
BMI	1.01	0.957	1.071	0.66
Course of study				
Medical	Ref			
Allied health	0.61	0.409	0.917	0.02
Academic year (final)				
Less than final	Ref			
Final	1.66	1.126	2.406	0.01
Vitamin D knowledge	1.03	0.983	1.073	0.24
Sun avoidance measures				
None	Ref			
At least one	0.91	0.723	1.140	0.41

The variables with p-values <0.1 in univariate regression (gender, academic year, and course of study) were considered in the multivariate model (Table 4). The odds of having low outdoor time were 1.46 higher for the female sex and final-year undergraduates. In addition, the course of study (Medical) was associated with an increased likelihood of low outdoor time.

**Table 4:** Factors associated with outdoor time using multivariate logistic regression.

	Exp (B)	95% C. I for B		P-value
		Lower Bound	Upper Bound	
Sex (females)	1.66	1.073	2.562	0.02
Course of study (Allied health)	0.57	0.378	0.871	0.009
Academic year (Final)	1.63	1.110	2.393	0.01

Log-likelihood ratio  = 601.7, Nagelkerke *R*^2^  =  5%, *X*^2^  =  16.79

## Discussion

The study provides evidence for vitamin D knowledge and sun exposure practices among Sri Lankan healthcare undergraduates. Vitamin D knowledge was poor in the vast majority. Unsatisfactory vitamin D knowledge among medical students [12] and physicians [18] has also been reported in other parts of the world. In contrast, Costa-Fernandes et al. reported adequate knowledge among physicians in northwest London (UK) [19]. They demonstrated that the knowledge positively impacted the prescription of correct vitamin D doses. In our study, undergraduates’ vitamin D-related knowledge was particularly poor on recommended practices for optimum sun exposure, non-skeletal health benefits and risk factors for VDD (viz., dark skin). The undergraduates who were low in knowledge frequently practised sun avoidance measures.

A growing number of reports have demonstrated the diverse health-promoting benefits of a safe level of sun exposure, which outweigh the risk of adverse effects such as skin cancer [20]. Most South Asians have Fitzpatrick skin colour type IV or V [21]. As a result, unprotected skin can be exposed to the sun for one hour or more without sunburn, and the risk of skin cancer is low [22]; thus, a modest amount of UVB exposure may be appropriate to avoid vitamin D deficiency [23, 24]. However, the vast majority of the undergraduates in this study used at least one sun protection method. They cited cosmetic reasons (skin darkening) for sun avoidance, probably because fair skin is often considered beautiful, a symbol of social dignity among South Asians [25]. Only a minority used sunscreen but frequently stayed under a shade for protection. According to previous reports, seeking shade and wearing long sleeves were the most important determinants of vitamin D levels compared to other sun-avoidance behaviours (viz., wearing a hat and sunscreen) among US adults between 18–60 years [26].

A smaller number of undergraduates stayed outdoors for more than 30 minutes daily. According to Farrar et al., Asians with dark skin need sun exposure much more than that to produce adequate vitamin D [24]. Academic workload may be preventing the undergraduates from staying outdoors. It is even more plausible that they lack adequate time management skills to allocate time for non-academic activities [27].

Medical undergraduates had less outdoor time than the allied health undergraduates in our study. In general, medical studies are perceived as very competitive. It is associated with less time for leisure activities or social contacts, heavy coursework, and demand for schedules compared to allied health study courses [28, 29]. Final-year undergraduates had fewer outdoor activities than pre-final years. The possible reasons could be that final year training in medical and allied health courses is intense, they are hospital-based, and they attend clinical work for long hours compared to pre-final years. Thus, outdoor time becomes very limited during the day.

Clear recommendations on safe sun exposure practices are required to enhance undergraduates’ vitamin D-related knowledge, practices and, in turn, personal health. Such guidance must be focused and practical to suit the undergraduates’ coursework and lifestyle. In Sri Lanka, routine vitamin D supplementation is not practiced; even high-risk groups such as pregnant women and infants are not provided vitamin D supplements due to intense debate on this subject. Therefore, future surveys should explore the link between specific sun exposure behaviors and serum vitamin D levels in Sri Lankan healthcare undergraduates to strategize preventive action against vitamin D deficiency.

We identified several limitations related to the study methods. First, because of the study’s observational nature, the causality of factors associated with outdoor time cannot be confirmed. Second, the participants were limited to one institution, and the findings cannot be generalized to all healthcare undergraduates in the country. Third, not including variables such as skin colour and socio-economic status that may impact outdoor time is a limitation of our study. Fourthly, sun-avoidance practices and outdoor time were reported but not observed; therefore, reporting bias could exist. Yet, the validity of self-reported sun avoidance practices has been confirmed in previous reports [30]. Finally, we did not conduct a sensitivity analysis for categorizing sun avoidance practices.

## Conclusions

The healthcare undergraduates had low vitamin D knowledge and outdoor time while having increased sun avoidance behaviour. Their knowledge was reflected in sun exposure practices. Gender, course of study, and academic year appeared to affect outdoor time. Support and guidance should improve vitamin D knowledge and sun exposure habits that suit their academic work and lifestyle. Also, universities can actively promote positive sun exposure by organizing outdoor events.
